# Association between autism and schizophrenia: shared pathways and distinctions

**DOI:** 10.1192/j.eurpsy.2026.11154

**Published:** 2026-07-31

**Authors:** T. Gutierrez Higueras

**Affiliations:** Child and Adolescent Psychiatry, Servicio Andaluz de Salud, Jaén, Spain

## Abstract

**Introduction:**

Autism spectrum disorder (ASD) and schizophrenia (SCZ) are two different conditions, each with its own unique features and life course. ASD usually appears in childhood and is marked by challenges in social communication, along with patterns of restricted or repetitive interests and behaviors. For many, these traits remain relatively stable over time. Schizophrenia, on the other hand, more often emerges in adolescence or adulthood. It is characterized by experiences such as hallucinations and delusions, and is frequently linked to a gradual decline in daily functioning.

**Objectives:**

To determine the association between ASD and SCZ, and check the common biological or genetic roots, similarities in symptoms, and the important differences in how each condition appears, evolves, and shapes everyday life.

**Methods:**

A literature search was carried out in PubMed to identify systematic reviews and meta-analyses published in the last ten years using the terms “autism” AND “schizophrenia”. Titles and abstracts were checked to ensure relevance, and avoid duplication. Finally, full articles that directly examined the association between ASD and SCZ were selected for qualitative synthesis.

**Results:**

The prevalence of schizophrenia is significantly higher in people with ASD compared to the general population. The findings show that both ASD and schizophrenia SCZ share impairments in cognitive, neurological, genetic, and environmental domains, although with differences in their magnitude and nature. At the cognitive level, both present deficits in executive functions (working memory, response inhibition, cognitive flexibility, and planning), but these are generally more marked in schizophrenia. Impairment in processing speed is observed in both conditions, and is also more pronounced in SCZ. Genetically, both disorders are highly heritable and show some overlap in rare and common variants, including deletions or duplications such as 16p11.2, 22q11.2, or 3q29, which increase the risk of developing ASD and SCZ.

**Conclusions:**

The summary of the evidence suggests that ASD and SCZ share a strong common neurobiological and genetic basis, but with different developmental trajectories, evidenced by clinical, cognitive, and brain differences. Executive functions and social cognition emerge as areas of shared vulnerability, although the underlying mechanisms appear differential. Furthermore, rare genetic variants, such as duplications in 16p11.2, could represent autism subtypes with a higher risk of psychosis.

The lack of direct comparisons between ASD and SCZ, primarily in individuals with comorbidity, is an important point. Future research is recommended to adopt more homogeneous designs, include large samples, and conduct longitudinal follow-ups to better understand how cognitive, biological, and environmental factors interact in the relationship between autism and schizophrenia.

**Disclosure of Interest:**

None Declared

